# Comparative proteomic analysis of the effects of high-concentrate diet on the hepatic metabolism and inflammatory response in lactating dairy goats

**DOI:** 10.1186/s40104-016-0065-0

**Published:** 2016-02-06

**Authors:** Yongqian Duanmu, Rihua Cong, Shiyu Tao, Jing Tian, Haibo Dong, Yuanshu Zhang, Yingdong Ni, Ruqian Zhao

**Affiliations:** Key Laboratory of Animal Physiology & Biochemistry, Ministry of Agriculture, Nanjing Agricultural University, Nanjing, Jiangsu China; College of Veterinary Medicine, Northwest A & F University, Yangling, Shannxi China

**Keywords:** High concentrate diet, Lactating goats, Liver, Mitochondria, Proteomics

## Abstract

**Background:**

To understand the impact of feeding a high-concentrate diet to mid-lactating goats for a long time on liver metabolism and inflammatory response, two dimensional polyacrylamide gel electrophoresis (2-DE) and real-time PCR method were employed to detect proteins differentially expressed in liver and their mRNAs expression in goats fed high concentrate diet (HC) or low concentrate diet (LC). Twelve lactating dairy goats were randomly assigned to either a HC diet group (65 % concentrate of dry matter; *n* = 6) or a LC diet group (35 % concentrate of dry matter; *n* = 6) for 10 wk.

**Results:**

Twenty differentially expressed spots (≥2.0-fold changes) in the hepatic tissues were excised and successfully identified using MALDI TOF/TOF. Of these, 8 proteins were up-regulated, while the rest 12 proteins were down-regulated in HC goats compared to LC. Differential expressed proteins including alpha enolase 1 (ENO1), glutamate dehydrogenase 1 (GLUD1), glutathione S-transferase A1 (GSTA1), ATP synthase subunit 5β (ATP5β), superoxide dismutase [Cu-Zn] (SOD1), cytochrom c oxidase subunit Via (COX6A1) and heat shock protein 60 (HSP60) were further verified by real-time PCR and/or western blot at mRNA or protein expression level. Consistent with the 2-DE results, a significant decrease of β-actin protein expression and SOD enzyme activity was observed in liver of HC goats (*P* < 0.05), while ENO1 protein expression was significantly up-regulated in HC compared to LC goats (*P* < 0.05) . However, western blot analysis did not show a significant difference of hepatic HSP60 protein between HC and LC group, which did not match the decrease of HSP60 content detected by 2-DE analysis. Real-time PCR showed that glutathione S-transferase P1 (GSTP1) and SOD1 mRNA expression was significantly decreased in liver of HC goats, while cytochrom c oxidase (COX3) and ATPase 8 (ATP8) mRNAs expression were markedly increased compared to LC (*P* < 0.05). Gene Ontology (GO) analysis revealed that HC diet resulted in altered expression of proteins related to catalytic and mitochondrial metabolism in the liver, and may increase the stress response with up-regulating the expression of differentiation 14 (CD14) cluster and serum amyloid A (SAA) as well as C-reactive protein (CRP) in the liver.

**Conclusions:**

These results suggest that feeding high concentrate diet to lactating goats for 10 wk leads to the activation of the inflammatory response, and decreases the anti-oxidant capacity, and subsequently impairs the mitochondrial function in the liver.

**Electronic supplementary material:**

The online version of this article (doi:10.1186/s40104-016-0065-0) contains supplementary material, which is available to authorized users.

## Background

Feeding high concentrate diet is the main energy supply for meeting high milk production demands, especially during a transition period from the pregnant non-lactating to the lactating stage [1]. However, it’s well known that feeding high amount of concentrate diet is likely to result in fermented disorders in the rumen (such as SARA) as well as in the large intestine, which generally causes lower ruminal pH and a significant increase of lipopolysaccharide (LPS) [2, 3]. LPS accumulated in the digestive tract even can translocate into the peripheral circulation system and then trigger the inflammatory response [4, 5]. An increasing evidences show that stress response was observed in cattle and goat after fed high concentrate diets exhibiting higher level of acute phase proteins including serum amyloid A (SAA) and C-reactive protein (CRP) [4–6]. Our previous result also showed that genes expression involved in inflammatory response and nutrients metabolism in the liver were significantly altered in lactating goats after fed a high concentrate diet for 9 wk [7].

As an important defense organ, liver plays a vital role in keeping body homeostasis when facing the external or internal antigens invasion. In the view of anatomic location, liver is likely attacked by variety of hazards derived from the portal vein system. Recently, the inflammatory response has been well documented in ruminants when they suffered SARA. Endotoxemia is usually caused by the translocation of LPS into the peripheral circulation through the damaged rumen epithelium in SARA status [8, 9], and then leading to the systemic inflammatory response [4]. In addition, it’s reported that LPS challenge can induce hepatic oxidative injury via decreasing of superoxide dismutase (SOD) enzyme activity and glutathione concentration (GSH) [10, 11]. A large group of genes expression involved in substrates metabolism were down regulated in liver of cattle after LPS challenged [12]. In ruminants, the liver is the major site for gluconeogenesis and lipogenesis, which provides the substrates precursors to the mammary gland for milk production. However, the hepatic metabolic process and the relative mechanisms are largely unknown in ruminants after fed HC diets for a long time.

Two dimensional polyacrylamide gel electrophoresis (2-DE) is considered as a powerful tool to identify significant global protein expression changes in response to different biological challenges [13]. The aim of this study was: 1) to determine global protein expression changes in the liver of lactating goats after fed a HC diet for a long time, and mainly focused on the hepatic inflammatory response and mitochondrial metabolism; and 2) to identify some preliminary molecular biomarkers for laying a better understanding the risk of disease. Our data may provide valuable hints for uncovering the negative effects of feeding high proportion of concentrate diet on hepatic metabolism and immune response.

## Methods

### Animals

The experiment design was presented in our previous study [14]. In brief, twelve healthy multiparous mid-lactating goats (Guanzhong dairy goats, Xi’an City, China) with an average initial BW of 49.7 ± 5.5 kg (mean ± SD) were housed in individual stalls in a standard animal-feeding house in a pasture for 12 wk including 2 wk of diet adaptation. Animals were randomly allocated to two groups: one group received a diet with low concentrate (35 % dry matter, LC, *n* = 6) and the other group received a high concentrate diet (65 % dry matter, HC, *n* = 6). The goats were given free access to fresh water throughout the experiment.

The experiment was conducted following the guidelines of the Animal Ethics Committee at Nanjing Agricultural University, China. The details of the Animal Ethical Treatment were described as the same in our previous publication [14].

### Samples collection

After 10 wk feeding, goats were slaughtered after overnight fasting. After slaughter, liver tissues were carefully removed and frozen immediately in liquid nitrogen, and then used for extracting RNA and proteins.

### Real-time PCR and western blotting

Total RNA was extracted from liver tissues using Trizol reagent (15596026, Invitrogen) according to the manufacturer’s protocols. Reverse transcription (RT) was performed using the total RNA (2 mg) in a final volume of 25 mL containing 1× RT-buffer, 100 U reverse transcriptase, 8 U RNase inhibitor (Promega, USA), 5.3 mmol/L random hexamer primers and 0.8 mmol/L dNTP (TaKaRa, Dalian, China). After incubation at 37 °C for 1 h, the reaction was terminated by heating at 95 °C for 5 min and quickly cooling on ice. Real-time PCR was performed using an Mx3000P (Stratagene, USA). Mock RT and No Template Controls (NTC) were set to monitor the possible contamination of genomic DNA both at RT and PCR. Two microliter of 20-fold diluted RT product was used for PCR in a final volume of 10 μL. Three technical replicates were analyzed for each biological replicate, and goat β-actin mRNA was used as a reference gene for normalization purposes. The PCR protocol began with an initial denaturation (1 min at 95 °C) followed by a three-step amplification program (20 s at 95 °C, 20–30 s at 60–62 °C, and 30 s at 72 °C), which was repeated 45 times. The primers sequences are shown in Table 1.

**Table 1:** The primer sequence of the target gene

Target genes	Primer	Primer sequence (5'→3')	Product length, bp	Accession No.
*SOD1*	Forward	CCAGTGCAGGTCCTCA	248	NM_001285550.1
	Reverse	AGCGTTGCCAGTCTTT		
*ENO1*	Forward	CCTGGAGAACAAAGAAGC	116	NM_174049.2
	Reverse	TGCCCGACCTGTAGAA		
*GLUD1*	Forward	CAACGGACCGACAACT	128	XM_005699318.1
	Reverse	GATTTAGATTATTCAGCCAC		
*PDIA3*	Forward	ACACGGGCTCTTCTGG	168	NM_001285732.1
	Reverse	CCTTTGCTAACGGGAC		
*GSTA1*	Forward	CCAACTTCCCTCTGCT	125	BC102540.1
	Reverse	CCTGGCTTCTTCTATTTT		
*HSP60*	Forward	GGCTCCTCATCTCACT	135	JF412686.1
	Reverse	ATCACTGTCCTTCCCT		
*AGMAT*	Forward	GGAGGCAGACCCATTT	153	XM_005690874.1
	Reverse	GTCACAACCCACCACATT		
*ACAT2*	Forward	AAAAGCAGGGTGGTCG	126	XM_005684985.1
	Reverse	GCTCCTCCTTCAGTGTT		
*COX6A1*	Forward	ATGAAGTCGCACCACG	117	GAAI01003433.1
	Reverse	AGGGTTATGGAATAGGGTA		
*CRP*	Forward	CTGGCTTGGGAGATTG	134	NM_001144097.1
	Reverse	AGTGAGGGTAAGGGATT		
*SAA*	Forward	CATCCTGCGTCTGGACCTGG	121	AF540564.1
	Reverse	TTCCTTGATGTCACGGACGATTT		
*HP*	Forward	TAATGCCCATCTGCCTAC	162	XM_004015111.1
	Reverse	CGCCCTCATAGTGTTTCA		
*LBP*	Forward	CAAGTAACAAGCCGGTAGCCC	138	XM_004014566.1
	Reverse	CCTGAAGAGGACCTGCGAGTAG		
*TNF-α*	Forward	CAAGTAACAAGCCGGTAGCCC	173	AF276985.1
	Reverse	CCTGAAGAGGACCTGCGAGTAG		
*IL-1β*	Forward	GAAGAGCTGCACCCAACA	172	D63351.1
	Reverse	CAGGTCATCATCACGGAAG		
*CD14*	Forward	CCGTTCAGTGTATGGTTGCC	239	NM_001077209.1
	Reverse	TGCTTCGGGTCGGTGTT		
*TLR4*	Forward	GTTTCCACAAGAGCCGTAA	195	JQ342090.1
	Reverse	TGTTCAGAAGGCGATAGAGT		
*MYD88*	Forward	ACAAGCCAATGAAGAAAGAG	98	JQ308783.1
	Reverse	GAGGCGAGTCCAGAACC		
*GSTA2*	Forward	ACTACTTGCCACCAAATACACC	90	XM_005696422.1
	Reverse	TCAAATGCAGGGAAATAACG		
*GSTP1*	Forward	AGACCTCACGCTGTACCAGTC	80	AF186248.1
	Reverse	CCTTCACATAGTCCTCCTTGC		
*GSTM1*	Forward	GCCATCCTTCGGTACATCG	90	AF249588.1
	Reverse	GCCAAGCGGACATCCATAA		
*COX1*	Forward	CATCGGCACCCTCTACCT	197	KP662714.1
	Reverse	AGGCTAACGTATGTGGGT		
*COX2*	Forward	GTAGAAACGGTCTGAACT	112	KP662714.1
	Reverse	TTACTGTGAGGGATGGGT		
*COX3*	Forward	TACTTAATACCTCCGTCCTC	175	KP662714.1
	Reverse	GAAATTGTGAATGGTGCTT		
*ATP6*	Forward	TCACAGCAGGACACTTAC	173	KP662714.1
	Reverse	GTATAGGCTGACTAGGAGA		
*ATP8*	Forward	CACATCGACATGACTTAC	102	KP662714.1
	Reverse	AGTTCTGGGTTGTGGTAG		
*GAPDH*	Forward	GGGTCATCATCTCTGCACCT	180	HM043737.1
	Reverse	GGTCATAAGTCCCTCCACGA		

Western blotting was used to validate the main differentially expressed proteins and carried out using a tank system (Bio-Rad). Frozen liver tissues (100 mg) were minced and homogenized in 1 mL of ice-cold homogenization buffer RIPA containing the protease inhibitor cocktail Complete EDTA free (Roche, Penz-berg, Germany). The homogenates were centrifuged at 12,000 rpm for 20 min at 4 °C and then collected the supernatant fraction. The protein concentration was determined with Pierce BCA Protein Assay Kit (catalog no. 23225, Thermo Scientific, Hudson, NH). Equal amount of proteins (20 ~ 50 μg) were subjected to 10 % SDS/PAGE gels according to the protein molecular weight and separated by electrophoresis, transferred to 0.45 μm pore-size nitrocellulose filter membranes (Bio Trace, Pall Co., USA). After transferred, membranes were blocked for 2 h at room temperature in blocking buffer and then membranes were incubated with the following primary antibodies: enolase α (ENO1, catalog no. BS6209, Bioworld, USA; diluted 1:500), heat shock protein 60 (HSP60; catalog no. ab46798, Abcam, Cambridge, UK; diluted 1:1,000), β-actin (catalog no. KC-5A08, Changcheng, China; diluted 1:10,000) and glyceraldehyde 3-phosphate dehydrogenase (GAPDH; catalog no. AP0063, Bioworld Technology; diluted 1:10,000). Antibody to the reference protein tubulin (catalog no. AP0063, Bioworld, USA; diluted 1:10,000) was used for normalization purposes in the analysis. After several washes in Tris-Buffered-Saline with Tween (TBST), membranes were incubated with goat anti-rabbit horseradish peroxidase (HRP) conjugated secondary antibodies (1:10,000; Bioworld, USA) in dilution buffer for 2 h at room temperature. Finally, the blot was washed, and bands were detected through enhanced chemiluminescence (ECL) using LumiGlo substrate (Super Signal West Pico Trial Kit, Pierce, USA). ECL signals were recorded using an imaging system (Bio-Rad, USA) and were analyzed using Quantity One software (Bio-Rad, USA).

### Two dimensional gel electrophoresis (2DE)

#### Sample preparation

Liver tissues were homogenized in the extraction buffer (7 mol/L urea, 2 mol/L thiourea, 3 % CHAPS, 50 mmol/L dithiothreitol). The homogenates were centrifuged at 15,000 g for 30 min at 4 °C. The sample was stored at −80 °C until analysis.

### Electrophresis

Immobilized pH gradient (IPG) strips (17 cm, pH 3–10) were rehydrated for 13 h at 250 V with 1.5 mg of proteins extracted from eight liver tissues (*n*=4/group), respectively. Isoelectric focusing was performed at 250 V for 1 h, 1,000 V for 1 h and followed by linearly ramping to 10,000 V over 5 h and then holding at 10,000 V until 60,000 V-h was reached. Before the second dimension, the IPG strips were first equilibrated for 15 min in 3 mL equilibration buffer (6 mol/L urea, 30 % glycerol, 2 % SDS, 50 mmol/L Tris pH 8.8, 1 % (v/v) DTT and then in a second equilibration for 15 min in the same equilibration buffer except that DTT was replaced by 4 % iodoacetamide. Electrophoresis was run initially at 10 mA/strip for 30 min followed 25 mA/strip on 12 % polyacrylamide SDS gel until the bromophenol blue dye reached the bottom edge of the gel.

### Image analysis and protein identification

Gels were stained with Coomassie Brilliant Blue G250 and scanned with Molecular Imager (Versa Doc3000, Bio-Rad, USA). Data analysis was conducted by PDQuest 8.0 software (Bio-Rad, USA). Selected gel spots were manually excised and washed twice with MilliQ water. The digested proteins were air-dried and analyzed by using a 4800 MALDI-TOF/TOF Proteomics Analyzer (Applied Biosystems, USA). A protein spot digested with trypsin was used to calibrate the mass spectrometer, using the internal calibration mode. A mass range of 800–3500 Da was used. A combined search (MS plus MS/MS) was performed using GPS Explorert TMsoftware v3.6 (Applied Biosystems, USA) and the MASCOT search engine (Matrix Science Ltd., UK). Protein identification was assigned when there were at least 4 matching peptides and >20 % sequence coverage.

### Gene ontology analysis

In this study, the function, biological process and cellular components of the differentially expressed proteins were analyzed according to the gene ontology (geneontology.org). This data could provide an overview of the main biological processes of these differentially expressed proteins involve in.

### Analysis of total anti-oxidative capacity and SOD activity

Total anti-oxidative capacity of liver (catalog no. A015, Shanghai Enzyme-linked Biotechnology Co. Ltd, Shanghai, China) and hepatic SOD enzyme activity (catalog no. A001-3, Jiancheng, Nanjing, China) were analyzed by commercial kits. The procedures were performed according to the manufacturer’s instructions.

### Statistical analyses

All data are presented as the mean ± SEM. Statistical significance was assessed by the independent sample *t*-test using SPSS (SPSS version 11.0 for Windows; SPSS Inc., Chicago, IL, USA) software packages. The 2^-ΔΔCt^ method was applied to analyze the real-time PCR data. Differences were considered significant at *P* < 0.05. Numbers of replicates used for statistics are noted in the Tables and Figures.

## Results

### Global identification of differentially expressed proteins in the liver

**Fig. 1:**
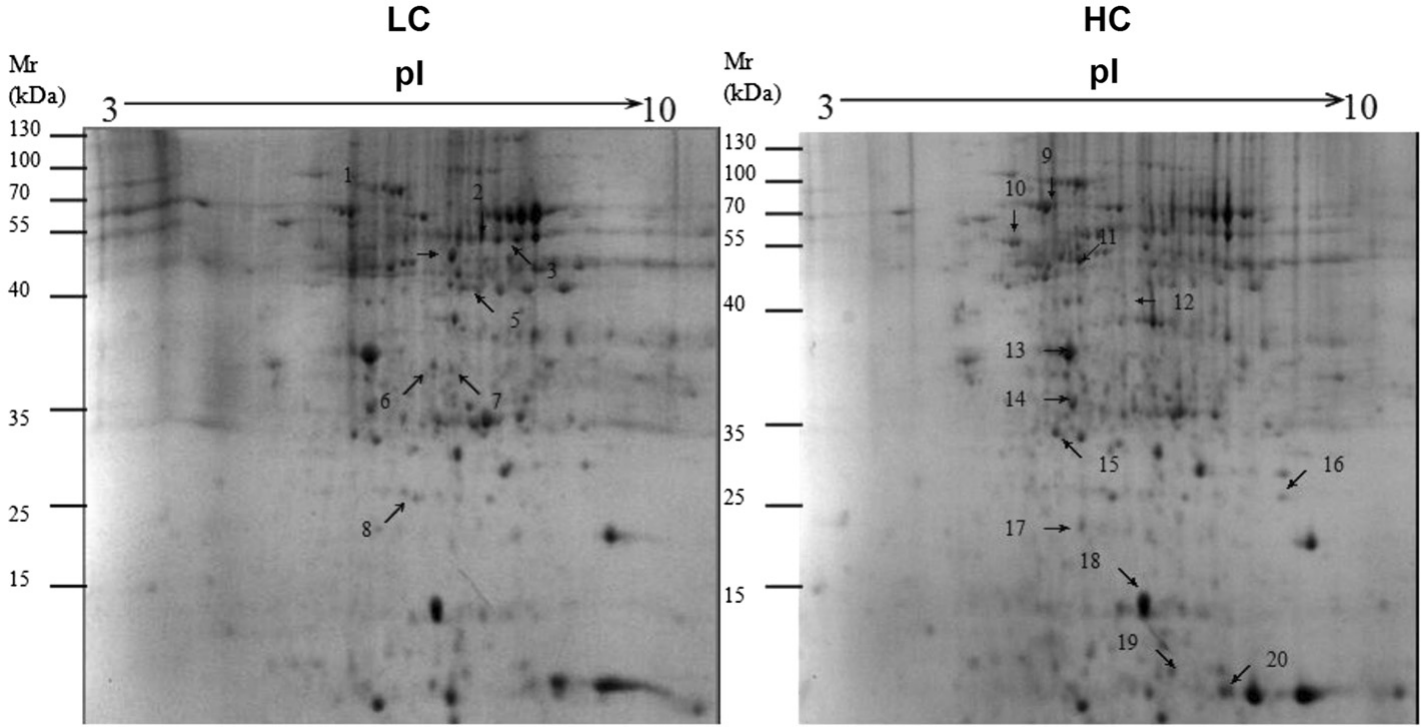
Differentially expressed proteins in liver of dairy goats fed HC and LC by two-dimensional gel electrophoresis analysis. The differentially expressed proteins between LC and HC goats were spotted and numbered. pI, isoelectric point; Mr, molecular mass; *n* = 4/group

To investigate the changes of protein expression profile in liver tissues between HC and LC goats, soluble proteins were analyzed by the 2-DE technique and followed with MALDI-TOF/TOF proteomics analyzer. Twenty differentially expressed proteins were analyzed and identified (Fig. 1, Additional file 1: Figure S1 ). Among these proteins, 8 proteins including glutamate dehydrogenase 1 (GLUD1, spot 3), UMP-CMP kinase-like isoform 1 (spot 4), aldehyde dehydrogenase, mitochondrial precursor (ALDH, spot 8), retinal dehydrogenase 1 (RALDH1, spot 9), agmatinase (AGMAT, spot 11), Acetyl-Coenzyme A acetyltransferase 2 (ACAT2, spot 12), alpha enolase (ENO1, spot 16) and 4-hydroxy-2-oxoglutarate aldolase, mitochondrial (HOGA1, spot 18) expression were up-regulated in the liver of HC goats compared to LC. However, the rest of 12 proteins including regucalcin (RNG, spot 1), Hemoglobin subunit beta-A (spot 2), glutathione S-transferase A1 (GSTA1, spot 5), ATP synthase subunit beta (ATPSβ, spot 6), superoxide dismutase [Cu-Zn] (SOD1, spot 7), cytochrom c oxidase subunit Via (COX6A1, spot 10), 60 kDa heat shock protein mitochondrial (Hsp60, spot 13), 3-hydroxyanthranilate 3,4-dioxygenase (HAAO, spot 14), albumin precursor (spot 15), protein disulfide-isomerase A3 precursor (PDIA3, spot 17), actin beta (spot 19) and retinol-binding protein (RBP, spot 20) was down-regulated in the liver of HC goats compared to LC (Table 2).

**Table 2:** Differentially expressed proteins in liver of HC and LC groups identified by two-dimensional gel electrophoresis analysis and MALDI-TOF-MS^a,b^

Spot number	Protein name	Accession number	Molecular weight, kDa	PI	Protein expression
Oxidative stress (4)					
1	Regucalcin (RNG)	gi|195972815	33,956	5.42	Down
7	Superoxide dismutase [Cu-Zn] (SOD1)	gi|27807109	15,844	5.85	Down
5	Glutathione S-transferase A1 (GSTA1)	gi|118151372	25,549	8.66	Down
17	Protein disulfide-isomerase A3 precursor (PDIA3)	gi|148230374	57,293	6.38	Down
Mitochondrial function (6)					
3	Glutamate dehydrogenase 1 (GLUD1)	gi|347300243	61,668	8.03	Up
6	ATP synthase subunit beta (ATPSβ)	gi|28461221	56,249	5.15	Down
8	Aldehyde dehydrogenase (ALDH)	gi|115496214	57,073	7.55	Up
10	Cytochrom c oxidase subunit Via (COX6A1)	gi|162817	9, 501	6.13	Down
13	60 kDa heat shock protein (HSP60)	gi|262205483	61,110	5.71	Down
18	4-hydroxy-2-oxoglutarate aldolase (HOGA1)	gi|73998495	38,052	7.6	Up
Metabolism (5)					
11	Agmatinase (AGMAT)	gi|194674166	39,419	8.77	Up
12	Acetyl-Coenzyme A acetyltransferase 2 (ACAT2)	gi|109659291	41,657	6.46	Up
14	3-hydroxyanthranilate 3,4-dioxygenase (HAAO)	gi|115495835	32,701	5.51	Down
16	Alpha enolase (ENO1)	gi|4927286	47,589	6.44	Up
4	UMP-CMP kinase-like isoform 1	gi|109087275	26,237	8.14	Up
RA metabolism (2)					
9	Retinal dehydrogenase 1 (RALDH1)	gi|57526379	55,417	6.37	Up
20	Retinol-binding protein (RBP)	gi|415585	15,473	5.89	Down
Transport protein (2)					
2	Hemoglobin subunit beta-A	gi|122540	16,068	6.75	Down
15	Albumin precursor	gi|1930850052	68,266	5.58	Down
Cytoskeletal structure (1)					
19	Actin,beta	gi|148744172	42,022	5.29	Down

^a^MALDI-TOF-MS = matrix-assisted laser desorption ionization-time of flight mass spectrometry. *pI* isoelectric point; *gi* GenInfo Identifier

^b^Protein name and accession numbers were derived from NCBI database

### Validation of differentially expressed proteins

**Fig. 2:**
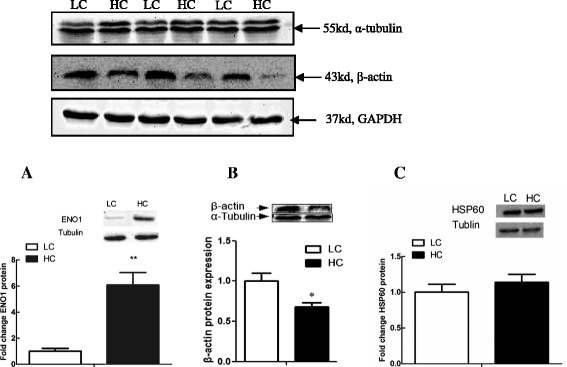
Western blot analysis of the ENO1, β-actin and Hsp60. **a** enolase α (ENO-1), **b** β-actin, **c** heat stress protein 60 (HSP60). Value is mean ± SEM. **p < 0.01 vs. LC. *n* = 6/group

**Fig. 3:**
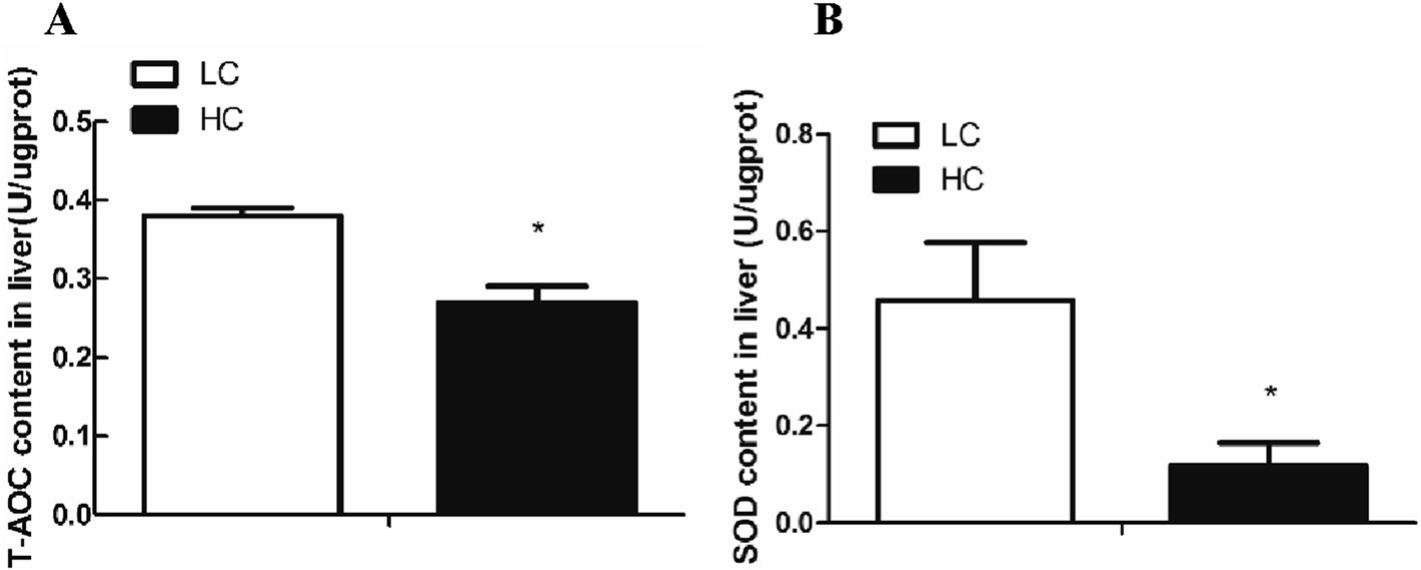
Comparison of total anti-oxidative capacity and SOD enzyme activity in liver. **a** Total anti-oxidant capacity (T-AOC), **b** SOD enzyme activity. The results are mean ± SEM. *P  < 0.05 vs. LC. *n* = 6/group

To confirm that the proteomic changes revealed by 2-DE method, the level of ENO1 (spot 7), β-actin (spot 19) and HSP60 (spot 13) proteins expression in the liver tissues was confirmed by western blotting analysis. As shown in Fig. 2, there was a good agreement between the results for 2-DE and western blot analysis. Hepatic ENO1 detected as a 47 kDa band was significantly up-regulated by high concentrate-fed diet, while β-actin protein was significantly decreased in liver of HC goats compared to LC. Moreover, the activity of SOD enzyme was significantly decreased in liver of HC goats, which was consistent with the down-regulation of its protein expression identified by 2D proteomics analysis (Fig. 3b). However, western blot result did not show a significant difference of hepatic HSP60 protein expression between HC and LC goats, which was not consistent with the analyzed result by 2-DE method.

**Fig. 4:**
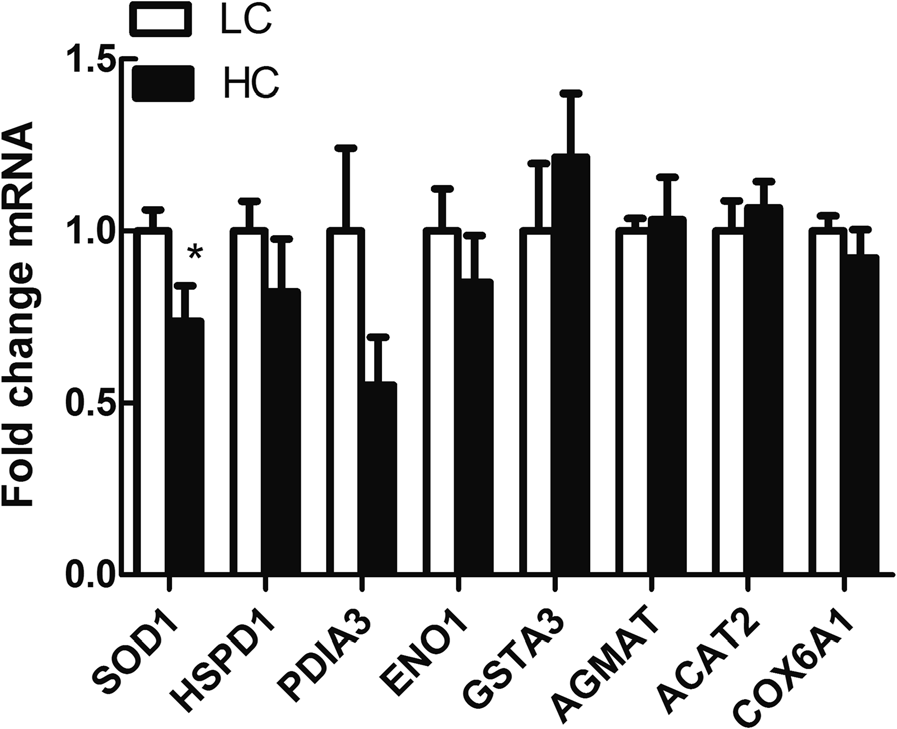
Real-time PCR analysis of the protein. Values are mean ± SEM. *P  < 0.05 vs. LC. GAPDH was used as the reference gene for gene expression. *n* = 6/group

In addition, in order to better understand the relationship of protein translation and genes expression, real-time PCR was used to analyze the level of genes expression encoding eight differentially expressed proteins (Fig. 4). The results showed that only SOD1 mRNA expression matched protein expression, showing a significant decrease in HC goats compared to LC (*P*  < 0.05). Protein levels do not necessarily correlate well with mRNA expression levels, and such discrepancies are often caused by the post-transcriptional regulation.

### Bioinformatics analysis of differentially expressed proteins

**Fig. 5:**
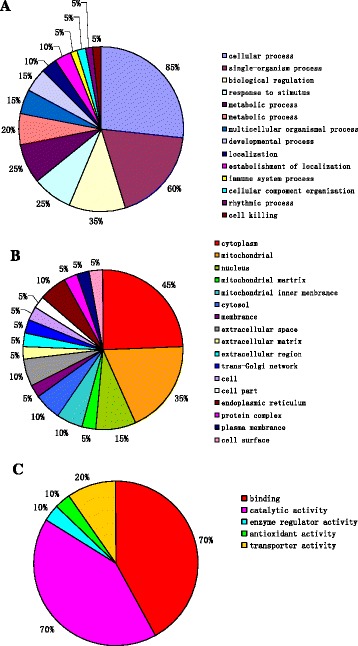
Gene ontology (GO) analysis of differentially expressed protein. **a** biological process **b** cellular components **c** molecular function

GO analysis is widely used in proteomic research to perform functional annotation of large protein sets identified by 2-DE. Proteins showing ≥ 2-fold differential expression were included in our GO analysis (Fig. 5). As shown in Fig. 5, three sets of ontology were classified. Seventeen proteins (70 %) were classified into catalytic activities and 17 proteins (70 %) as binding functions identified in the “molecular function” set. For “biological process” database, 85 % of identified proteins are involved in cellular process and 60 % of proteins are associated with single-organism process, and 35 % of proteins are involved in the biological regulation. It’s very important to note that in cellular components database, 9 proteins belong to cytoplasm proteins and 6 proteins play roles in mitochondrial metabolism.

### Real-time PCR analysis

**Fig. 6:**
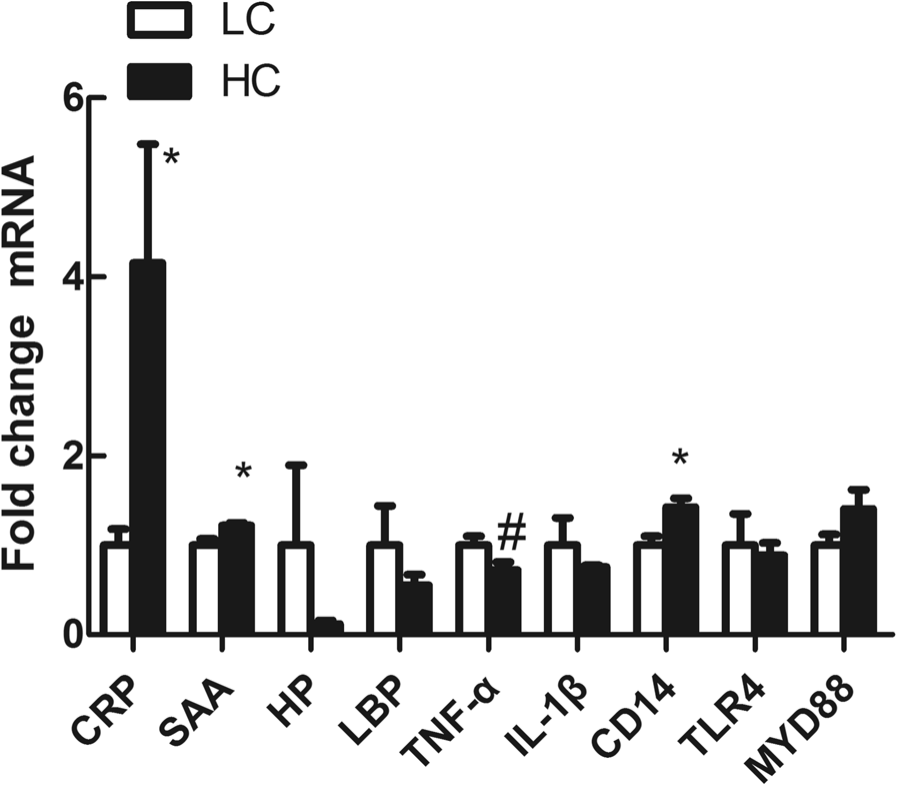
Acute phase response in liver induced by high concentrate diet. Values are mean ± SEM. *#p < 0.1* *P< 0.05 vs. LC. GAPDH was used as the reference gene for gene expression. *n* = 6/group

**Fig. 7:**
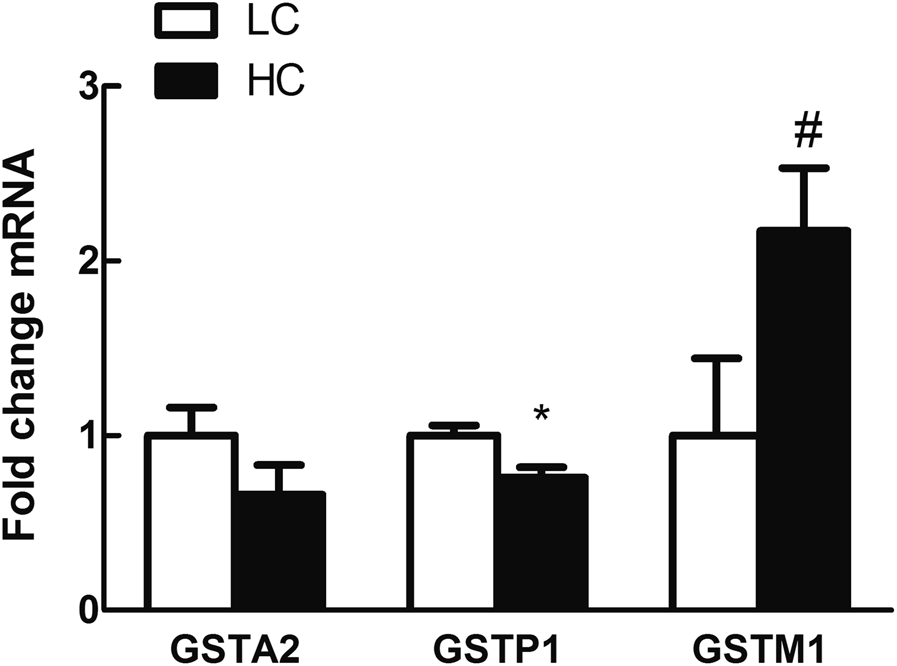
Real-time PCR analysis of GSTs family gene expression. Values are mean ± SEM. *#* *P*< 0.1 *p < 0.05 vs. LC. GAPDH was used as the reference gene for gene expression. *n* = 6/group

**Fig. 8:**
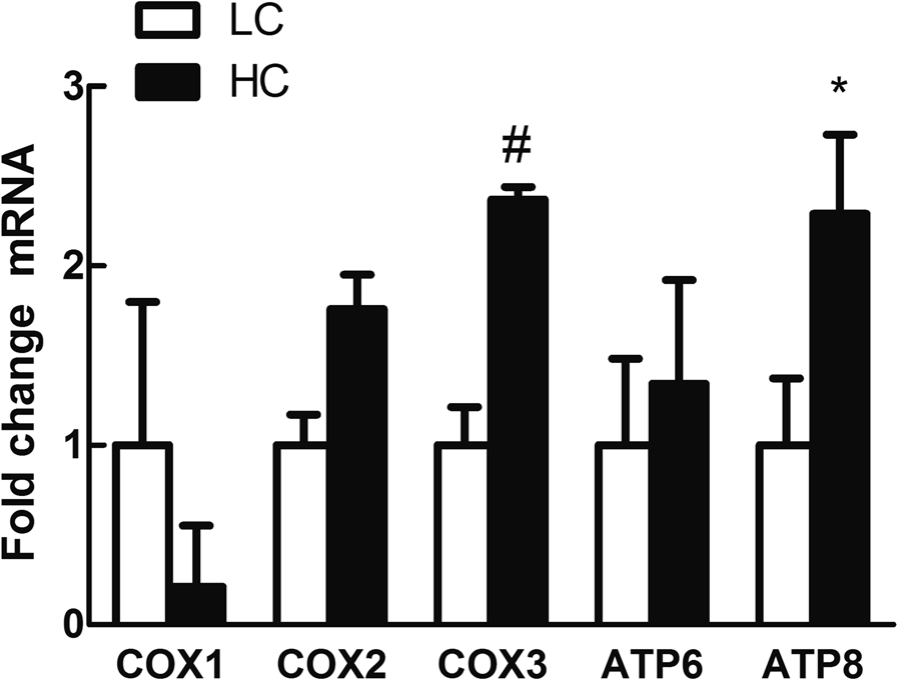
Gene expression of mitochondrial gene. Values are mean ± SEM. *#* *P < 0.1* *P < 0.05 vs. LC. GAPDH was used as the reference gene for gene expression. *n* = 6/group

Hepatic *CRP*, *SAA* and *CD14* mRNA expressions were significantly increased in HC goats compared to LC (*P < 0.05*), while *TNF-α* mRNA expression showed a potential decrease in HC goats (*0.05 < P < 0.1*) (Fig. 6). Among glutathione S-transferase (GST) family genes (Fig. 7), hepatic GSTP1 mRNA expression was significantly down-regulated (*P* < 0.05), yet GSTM1 gene expression showed a tendency to increase in the liver of HC goats compared to LC (*0.05 < P< 0.1*). In hepatic mitochondria, COX3 and ATPase 8 genes expression was potentially increased in HC goats (*0.05 < P < 0.1*) (Fig. 8).

### Total anti-oxidative capacity (T-AOC) and SOD enzyme activity

As shown in Fig. 3, the level of total anti-oxidative capacity (T-AOC) and SOD enzyme activity was significantly decreased in liver of HC goats compared to LC (*P*  < 0.05) (Fig. 3).

## Discussion

Previous researches have shown that feeding a high-concentrate diet to lactating dairy cows can cause inflammatory response in liver of ruminants [4, 8]. In this study, the differentially expressed proteins determined by 2-DE and MALDI-TOF/TOF are mainly involved in regulating oxidative stress, mitochondrial function and retinoic acid metabolism in liver of lactating goats fed a high concentrate diet for 10 wk.

### High-concentrate diet causes acute phase response and oxidative stress

It’s reported that high-concentrate challenge results in acute phase response indicating by higher level of acute phase proteins (APP) including SAA, Hp and LBP produced in the liver of dairy cows and even accompanied by the translocation of LPS from the digestive tracts into blood [4]. In this study, the transcriptional level of CD14, a glycoprotein that plays a crucial role for recognizing LPS in liver [15, 16], was significantly up-regulated in the liver of HC animals. It’s reported that in rat, LPS treatment *in vivo* can stimulate hepatic *CD14* gene expression [17]. In addition, we also found a significant increase of *CRP* and *SAA* mRNA expression in the liver of HC goats compared to LC, which was consistent with the previous studies [6, 7]. As a major site for synthesizing circulating inflammatory cytokines [18, 19], liver could control the homeostasis balance through responding to both endogenous and exogenous stimuli. However, except a slight decrease of *TNF-α* mRNA expression, there was no significant difference of cytokines genes expression in the liver of HC and LC goats. The increase of LPS in rumen as well as in the hindgut lumen was observed in the ruminants after fed a HC diet for a period [2]. In this study, no significant changes of hepatic cytokines genes expression observed in HC goats may indicate a high capacity of detoxification in liver and/or in the digestive tract, which still waits for further study. Nevertheless, to a certain extent, the activation of hepatic APP genes expression still indicates a stress status of lactating goats fed a high concentrate diet for 10 wk.

Regucalcin (RNG) is a regulatory protein in cell signaling system, which is considered to play a pivotal role in keep of cell homeostasis and function [20]. Over-expression of RNG can suppress cell death and apoptosis in rat [21]. In addition, regucalcin has a stimulatory effect on SOD expression in the liver cytosol of rats, and then benefits hepatic cells function in the body [21]. On the contrary, down-regulation of RNG may led to the decrease of SOD activity, and thereby dysfunction the antioxidant defense [21, 22]. Here, we found a decrease of total anti-oxidant capacity (TAOC) in liver of HC goats, which was consistent with the study conducted by Guo et al. [23].

### High-concentrate diet causes mitochondrial dysfunction

According to Gene ontology analysis, the level of 6 proteins involved in mitochondrial function was altered in liver by HC diet. GLUD1 could be considered as a sensitive marker of hepatotoxicity, which is highly expressed in the hepatic mitochondria. The up-regulation of GLUD1 indicated the mitochondria dysfunction [24, 25]. HSP60 is a mitochondrial chaperonin involving in the catalysis of proteins folding destined for the matrix, and maintaining proteins in an unfolded state to facilitate their function [26]. Recently, it’s reported that overexpression of Hsp60 failed to protect cells from oxidative stress due to a lack of its mitochondrial retention [27, 28]. Taken together, the changes of hepatic GLUD1 and HSP60 may lead to the increase of ROS and radical levels and then cause the mitochondrial dysfunction. In addition, the major function of ALDH2 is catalyzes the oxidation of aldehyde (e.g. products of lipid peroxidation) to carboxylic acids [29]. The loss of ALDH2 enzyme activity leads to the increase of mitochondrial oxidative stress [30]. COX6a1,which exerts a protective effect against ROS-induced cell damage [31], may contribute to the formation of an interaction site for cytochrome c [32]. Down-regulation of Cox6a1 indicates an unstable stability of holoenzyme. Therefore, the down-regulation of HSP-60, ATPSβ, COX6a accompanied with the up-regulation of GLUD1, ALDH and oxoglutarate aldolase indicate the mitochondrial dysfunction in HC goats compared to LC counterparts.

### High-concentrate diet changes hepatic metabolism

ENO1 is the glycolysis enzyme that catalyzes the production of phosphoenolpyruvate from 2-phosphoglycerate. Although ENO1 and mitochondrial dysfunction connection is not clearly, mitochondrial oxidative stress could induce glycolysis [33]. Jiang et al. reported that feeding 60 % HC diet to lactating dairy goats for 8 wk caused an up-regulation of ENO1 protein level in liver [34]. RALDH1 is a crucial enzyme that catalyzes the retinal to retinoic acid (RA) which exerts the anti-inflammation effects [35]. Rezamand et al. (2012) reported that the expression of RBP was positively correlated with TNF-α level in bovine liver [36]. In this study, the consistence of a down-regulation of RBP protein identified by 2D analysis and a mild decrease of TNF mRNA expression in liver of HC goats may support the positive relationship of RBP and TNF-α, which still needs for further investigation. In this study, a significant decrease of SOD1 mRNA expression and protein translation as well as lower activity of SOD enzyme was observed in liver of HC goats indicating the decline of anti-oxidant capacity of liver tissues. Consistently, the decrease of anti-oxidant capacity was also confirmed in HC goats by the index of total anti-oxidant capacity (T-AOC) determined by biochemical analysis (Fig. 3). Moreover, it’s very interesting to note that the expression of ENO1 and HSPD1 mRNA was not correlated with their proteins translation in liver indicating a post-transcriptional regulation involved in the regulatory process, such as miRNAs, which still needs further study.

## Conclusions

In conclusion, these results suggest that feeding 65 % HC diet to lactating goats for 10 wk leads to the activation of the inflammatory response, and decreases the anti-oxidant capacity as well as the dysfunction of mitochondrial metabolism in liver.

## Additional file

Additional file 1:
**Supplementary information. Figure S1.** Differentially expressed proteins in liver of dairy goats fed HC and LC by two-dimensional gel electrophoresis analysis. (DOCX 421 kb)
