# Supplementary material for: Comparative proteomic analysis of the effects of high-concentrate diet on the hepatic metabolism and inflammatory response in lactating dairy goats
Source: J Anim Sci Biotechnol. 2016 Feb 6;7:5. doi: 10.1186/s40104-016-0065-0 (PMC4744397; doi:10.1186/s40104-016-0065-0)
Supplement: Additional file 1: — Supplementary information. Figure S1. Differentially expressed proteins in liver of dairy goats fed HC and LC by two-dimensional gel electrophoresis analysis. (DOCX 421 kb) [file 40104_2016_65_MOESM1_ESM.docx]

**Supplementary information**

**Figure S1. Differentially expressed proteins in liver of dairy goats fed HC and LC by two-dimensional gel electrophoresis analysis.**

The differentially expressed proteins between LC and HC goats were spotted and numbered. pI, isoelectric point; Mr, molecular mass; Animals no. LC 2-4, HC 2-4.


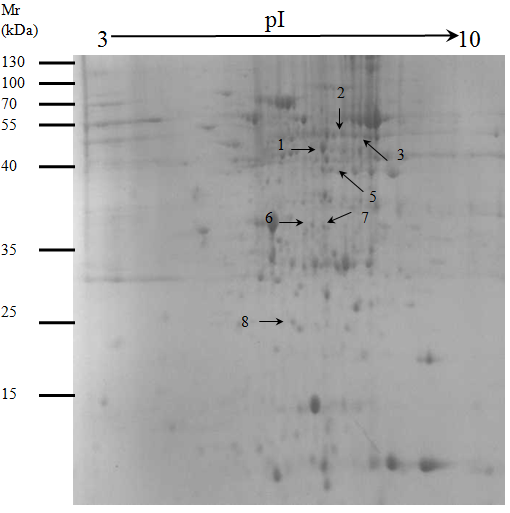

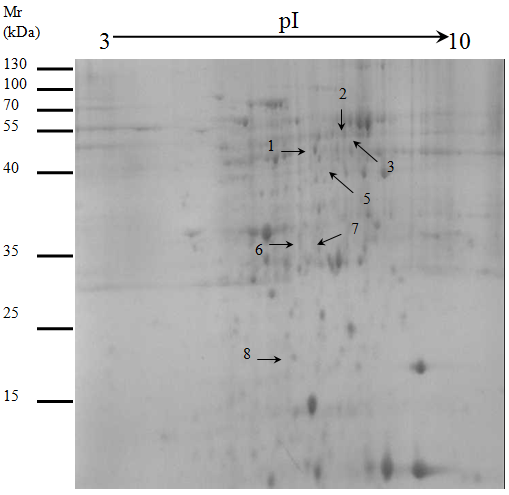

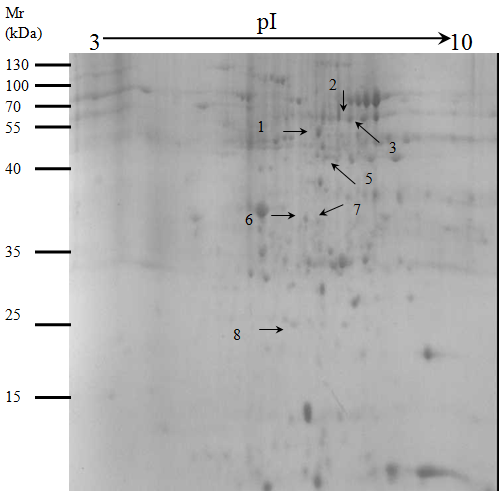

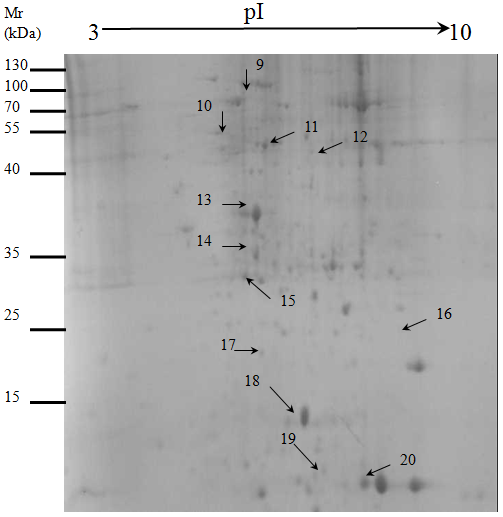


**LC-2**

**LC-3**

**HC-2**

**LC-4**


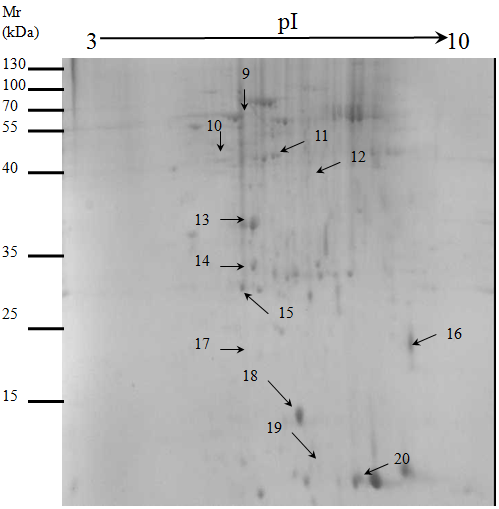

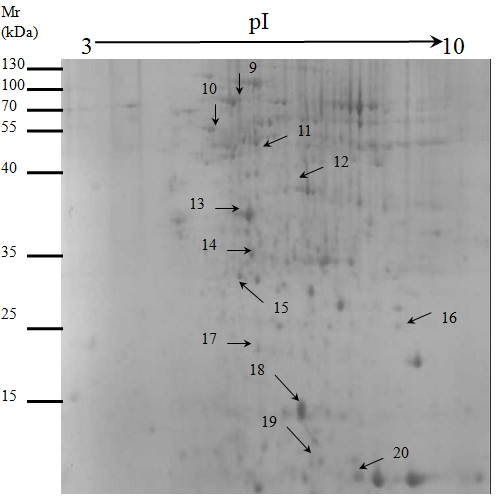


**HC-4**

**HC-3**
